# Quantifying Intracellular Distributions of HaloTag-Labeled Proteins With SDS-PAGE and Epifluorescence Microscopy

**DOI:** 10.21769/BioProtoc.5391

**Published:** 2025-07-20

**Authors:** Julia Shangguan, Ronald S. Rock

**Affiliations:** 1 Department of Biochemistry and Molecular Biology, University of Chicago, Chicago, IL, USA; 2 The Institute for Biophysical Dynamics, University of Chicago, Chicago, IL, USA

**Keywords:** Protein quantitation, Microscopy, Fluorescence, Protein localization, HaloTag, Myosin, Image analysis

## Abstract

Counting protein molecules helps reveal the organization of components within cellular structures and the stoichiometries of protein complexes. Existing protein and peptide quantitation methods vary in their complexity. Here, we report a straightforward workflow to measure the absolute number of HaloTag-labeled myosin 10 (Myo10) molecules in U2OS cells. Myo10 is a motor protein that plays a prominent role in cellular protrusion formation. Various biochemical and biological properties of Myo10 are established, but it is not well-defined how many molecules of Myo10 pack into narrow cellular structures called filopodia. We present a workflow for using SDS-PAGE to calibrate Myo10 signal with a reference protein, segmenting epifluorescence microscopy images to map Myo10 intracellular distribution, and interpreting the results to derive biological and functional insights. Our protocol is simple to employ and not only applicable for Myo10 research but also easily adaptable for other biological systems that use HaloTag.

Key features

• Combining SDS-PAGE densitometry with epifluorescence microscopy to quantitate HaloTag-labeled proteins in cells with readily available equipment.

• Details for quantifying protein signal intensity in cellular compartments with semi-automated image segmentation.

## Graphical overview


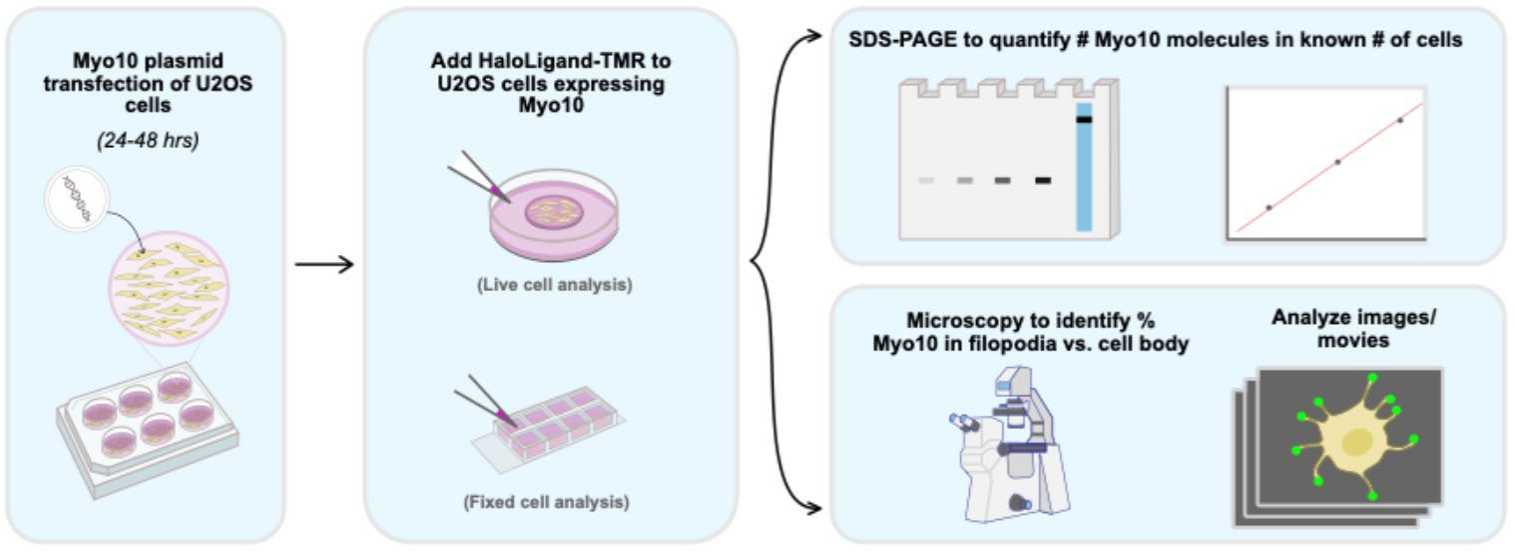


## Background

Over 40 classes of unconventional myosins exist [1], one of which is myosin 10 (Myo10). Myo10 is an unconventional motor protein that contributes to cellular protrusion formation [2]. Walking on bundled parallel actin filaments in structures called filopodia, Myo10 binds proteins and lipids to effect important processes such as cellular adhesion, environmental sensing during migration, and axon guidance [3]. When Myo10 is overexpressed or dysregulated, there are negative health consequences. Upregulated Myo10 has been tied to cancer progression through mechanisms of invasive protrusions and genomic instability [4–6].

Because of Myo10’s importance in proper biological function, past groups have characterized multiple aspects of its mechanism. This includes studies on Myo10 velocity [7], filopodial length [8], filopodial number [9], stepping behavior [10], actin selectivity [11], binding partners [12], and domain mutations [9,13]. Microscopy images display Myo10’s dense localization in filopodial tips, but there has not been extensive quantification of Myo10 distributions within the cell. Therefore, we recently developed a straightforward imaging and analysis method using HaloTag labeling technology to analyze the number of Myo10 molecules in filopodia [14].

There are various established approaches for counting proteins in cells [15–18]. Indeed, there are previous studies that count motor proteins using live-cell confocal microscopy [19] and super-resolution microscopy [20]. A drawback to methods using super-resolution microscopy [20,21] and mass spectrometry [22] is their requirement for special instrumentation that is not readily accessible. Instead, we share a strategy similar to quantitative western blotting of GFP [18] that is less time-consuming and does not rely on antibody binding. While alternative approaches such as single-molecule counting can offer high sensitivity, they rely on single-point calibrations and are more accurate when quantifying small numbers of molecules. These methods are less reliable when extrapolating from one molecule to tens of thousands. Furthermore, single-molecule validation typically depends on identifying stepwise photobleaching behavior to confirm that only one fluorophore is present. To avoid these technical complexities, our method utilizes HaloTag labeling and common tools, requiring only epifluorescence microscopy and SDS-PAGE.

Here, we outline detailed steps for preparing, staining, and analyzing cellular images of protein distributions in filopodia that are compatible with fixed and live cell samples. This protocol can be used to examine changes in Myo10 abundance and distribution in new contexts, but is also advantageous for any biological system with HaloTag labeling.

## Materials and reagents

**Biological materials**

1. HaloTag-Myo10-Flag plasmid (generated in-house via Gibson Assembly), purified from DH5 Alpha *E. coli* cells (Zymo Research, catalog number: T3007). Plasmid contains N-terminal Halotag (Promega; GenBank: JF920304.1), full-length human Myo10 sequence (nucleotide sequence from GenBank: BC137168.1), and C-terminal Flag-tag (GATTATAAAGATGATGATGATAAA). See File S1 for full nucleotide sequence

2. Human calmodulin 1 plasmid (Origene, catalog number: SC115829)

3. U2OS cells (ATCC, catalog number: HTB-96)

**Reagents**

1. Anti-myosin10 antibody (rabbit polyclonal) (Novus, catalog number: NBP1-87748)

2. Anti-beta-tubulin antibody (mouse monoclonal) (Invitrogen, catalog number: 22833)

3. Anti-rabbit-HRP antibody (goat polyclonal) (Cell Signaling, catalog number: 7074)

4. Anti-mouse-HRP antibody (horse polyclonal) (Cell Signaling, catalog number: 7076)

5. Laminin (Sigma-Aldrich, catalog number: CC095-M)

6. HaloTag standard protein (Promega, catalog number: G4491)

7. SuperSignal West Femto chemiluminescent substrate (Thermo Scientific, catalog number: 34094)

8. Gibco 1× DMEM (Thermo Fisher, catalog number: 11995073)

9. Fetal bovine serum (FBS) (Cytiva, catalog number: SH30071)

10. Gibco Opti-MEM I reduced serum medium (Thermo Fisher, catalog number: 31985062)

11. Accutase enzyme cell detachment solution (Corning, catalog number: MT25058CI)

12. FuGENE HD transfection reagent (Promega, catalog number: E2311)

13. Lipofectamine 2000 (Invitrogen, catalog number: 11668-027)

14. #1.5 coverglass bottom 35 mm Petri dishes, 10 mm micro-well (Cellvis, catalog number: D35-10-1.5-N)

15. Ibidi 8-well chambered coverslip (Ibidi, catalog number: 80807)

16. 6-well Nunc cell culture–treated dish (Thermo Fisher, catalog number: 140675)

17. 4%–20% Mini-PROTEAN TGX stain-free protein gel (Bio-Rad, catalog number: 4568095)

18. TMR-HaloLigand, 5 mM (Promega, catalog number: G8251)

19. Alexa Fluor 647 Phalloidin, 300 units (Invitrogen, catalog number: A22287) (300 units dissolved in 150 µL DMSO, as per manufacturer’s recommendation)

20. Bio-Beads SM-2 (Bio-Rad, catalog number: 152-8920)

21. Protease cocktail inhibitor (Sigma, catalog number: P8849)

22. BLUEstain 2 protein ladder, 5–245 kDa (GOLDBIO, catalog number: P008-500)

23. Bovine serum albumin (BSA) (Sigma-Aldrich, catalog number: A9647)

24. Instant nonfat dry milk (Stop & Shop)

25. Sterile deionized water

26. PIPES 99.5%, BPC tested (Sigma-Aldrich, catalog number: P1851)

27. EGTA (Research Products International, catalog number: E57060-50.0)

28. Magnesium chloride hexahydrate (MgCl_2_·6H_2_O) (Fisher Scientific, catalog number: BP214500)

29. 16% paraformaldehyde aqueous solution, EM grade (PFA) (Fisher Scientific, catalog number: 50-980-487)

30. Triton X-100 (Fisher Scientific, catalog number: 64-846-410)

31. Potassium chloride (Sigma-Aldrich, catalog number: P9541)

32. Imidazole (Fisher Scientific, catalog number: AC301870010)

33. DTT (Thermo Scientific, catalog number: R0861)

34. Catalase from bovine liver (Sigma-Aldrich, catalog number: C3155)

35. Glucose oxidase (Gold Biotechnology, catalog number: G-041-100)

36. Tris base (Fisher Scientific, catalog number: bp1521)

37. Sodium chloride (Fisher Scientific, catalog number: S2711)

38. Tergitol solution, type NP-40 (Sigma-Aldrich, catalog number: NP40S)

39. Sodium deoxycholate (Fisher Scientific, catalog number: AAJ6228814)

40. 10% SDS solution (Fisher Scientific, catalog number: BP24361)

41. Glycine (DOT Scientific, catalog number: DSG36050-1000)

42. Tricine (Millipore Sigma, catalog number: T0377)

43. EDTA (Fisher Scientific, catalog number: 327205000)

44. Methanol (Fisher Scientific, catalog number: A412-4)

45. DMSO, anhydrous (Thermo Scientific, catalog number: D12345)

46. PMSF (Gold Biotechnology, catalog number: P-470-10)

47. PBS, no calcium, no magnesium (Thermo Scientific, catalog number: 14190136)

48. DAPI (Thermo Scientific, catalog number: 62248)

49. Glycerol (Millipore Sigma, catalog number: G9012)

50. Bromophenol blue (Thermo Scientific, catalog number: A18469.18)

51. Trypan blue solution, 0.4% (Thermo Scientific, catalog number: 15250061)

52. Tween 20 (Sigma-Aldrich, catalog number: P6585)

**Solutions**

1. Laminin coating (see Recipes)

2. PEM buffer (see Recipes)

3. Fixation and staining solution (see Recipes)

4. 1% BSA blocking solution (see Recipes)

5. 10× AB buffer (see Recipes)

6. 50× GOC (see Recipes)

7. RIPA lysis buffer (see Recipes)

8. Semi-dry transfer buffer (see Recipes)

9. 5× SDS loading buffer (see Recipes)

**Recipes**

**1. Laminin coating**

Aliquot the laminin stock solution (20 µL for a manufacturer’s stock of 2 mg/mL; adjust accordingly) in 2 mL Eppendorf tubes under the tissue culture hood and freeze at -20 °C. On the day of seeding cells on a coverslip, hand-thaw a laminin aliquot vial and add PBS for a final concentration of 20 µg/mL. Gently vortex to mix, then directly pipette 170 µL to each well of the 8-well chambered coverslip or #1.5 coverglass-bottom 35 mm Petri dish with a 10 mm micro-well (round coverslip), ensuring the entire surface area is covered. Incubate the coverslip in the tissue culture incubator for 15–20 min. Aspirate the laminin coating (leaving a tiny film of liquid behind) before seeding cells. Seed cells suspended in DMEM + 10% FBS for 50%–60% cell density on glass. Laminin coating solution can be stored at 4 °C for one week.

**Table:** 

Reagent	Final concentration	Volume
Laminin	20 µg/mL	20 µL (if stock is 2 mg/mL)
PBS	n/a	1,980 µL

**2. PEM buffer (pH 6.95, 0.2 µm filtered, 500 mL)**

PIPES is light-sensitive, so store PEM buffer at 4 °C in the dark and use within 6 months.

**Table:** 

Reagent	Final concentration	Quantity or volume
PIPES	0.1 M	15.11 g
EGTA	5 mM	0.95 g
MgCl_2_·6H_2_O	2 mM	0.20 g
H_2_O	n/a	500 mL (final volume)

**3. Fixation and staining solution (2 mL)**

Prepare 2 mL to label all wells of an 8-well chambered coverslip. This should be kept on ice and in the dark.

**Table:** 

Reagent	Final concentration	Quantity or volume
16% PFA	4%	500 µL
10% Triton	0.08%	16 µL
5 mM TMR-HaloLigand	2.5 µM	1 µL
DAPI	1:1,000 dilution	2 µL
PEM buffer	n/a	1,481 µL

**4. 1% BSA blocking solution (0.2 µm filtered, 5 mL)**

This can be stored at 4 °C for two weeks.

**Table:** 

Reagent	Final concentration	Quantity or volume
BSA	1%	0.05 g
PBS	n/a	5 mL (final volume)

**5. 10× AB buffer (pH 7.5, 20 mL)**

This can be stored as 1 mL aliquots in -20 °C.

**Table:** 

Reagent	Final concentration	Quantity or volume
Potassium chloride	250 mM	0.37 g
Imidazole	250 mM	0.34 g
MgCl_2_·6H_2_O	40 mM	0.16 g
EGTA	40 mM	0.30 g
1 M DTT in H_2_O	1 mM	20 µL
H_2_O	n/a	20 mL (final volume)

**6. 50× GOC**

This is an enzymatic oxygen-scavenger system used in live-cell imaging experiments. GOC improves dye stability by reducing photobleaching.

**Caution:** The substrate oxidation reaction results in acidification of the buffer, so monitor cell health. GOC is not required for the live imaging experiments, though it could help improve dye signal. We have not observed a noticeable difference with or without GOC during live imaging.

Combine the catalase and glucose oxidase protein solutions 1:1 in a 3.5 mL thickwall polycarbonate tube, pipette up and down 5× with a 1,000 µL tip, and spin at 95k × *g* (using Optima Max-XP Tabletop Ultracentrifuge, Rotor TLA 100.3) for 15 min at 4 °C. Spinning removes precipitants in the catalase solution.

Keep the supernatant and mix with an equal volume of glycerol. This can be stored as 100 µL aliquots in -20 °C. If the GOC solution is frozen solid, it is unusable.

**Catalase solution**

**Table:** 

Reagent	Final concentration	Quantity or volume
Catalase	7.2 mg/mL	720 µL
10× AB buffer	2× AB	200 µL
H_2_O	n/a	80 µL
Total	n/a	1 mL

**Glucose oxidase solution**

**Table:** 

Reagent	Final concentration	Quantity or volume
Glucose oxidase	43.2 mg/mL	43.2 mg
10× AB buffer	2× AB	200 µL
H_2_O	n/a	800 µL
Total	n/a	1 mL

**50× GOC**

**Table:** 

Reagent	Final concentration	Quantity or volume
Glucose oxidase solution (post-spin)	4.3 mg/mL	1 mL
Catalase solution (post-spin)	0.7 mg/mL	1 mL
Glycerol	50%	2 mL
Total	n/a	4 mL

**7. RIPA lysis buffer (pH 7.4, 0.2 µm filtered, 250 mL)**

This can be stored at 4 °C for one week.

**Table:** 

Reagent	Final concentration	Quantity or volume
Tris base	50 mM	1.5 g
Sodium chloride	150 mM	2.2 g
NP-40	1%	2.5 mL
Sodium deoxycholate	0.5%	1.25 g
SDS	0.1%	250 µL
H_2_O	n/a	247.25 µL
Total	n/a	250 mL

**8. Semi-dry transfer buffer (pH 8.5, 1 L)**

No need to adjust pH. This can be stored at room temperature for at least two years.

**Table:** 

Reagent	Final concentration	Quantity or volume
Tris base	336 mM	40.7 g
Glycine	260 mM	19.5 g
Tricine	140 mM	25.1 g
EDTA	24 mM	0.9 g
H_2_O	n/a	1 L (final volume)

**9. 5× SDS loading buffer**

0.1 M DTT, 2% SDS, 0.05% bromophenol blue, 0.05 M Tris-HCl, 10% glycerol, pH 6.8

**Laboratory supplies**

1. 1.5 mL protein LoBind tube (Eppendorf, catalog number: 022431081)

2. 1.5 mL microcentrifuge tubes (Fisher Scientific, catalog number: 05408129

3. PVDF membrane (Millipore Sigma, catalog number: ISEQ00005)

4. Extra-thick western blotting filter paper (Thermo Scientific, catalog number: 88610)

5. 3.5 mL thickwall polycarbonate tube (Beckman Coulter, catalog number: 349622)

## Equipment

1. Biological Safety Cabinet Class II Type A2 (Nuaire, model: NU-540)

2. Epifluorescence microscope with 60×, 1.2 NA water objective and LED light source (Zeiss Axiovert 200)

3. Forma Series II Water Jacket CO_2_ incubator, set at 37 °C, 5% CO_2_ (Thermo Scientific, model: 3110)

4. Mini Protean II Electrophoresis System (Bio-Rad, model: 125BR)

5. NanoDrop One^c^ Microvolume UV-Vis Spectrophotometer (Thermo Scientific)

6. ChemiDoc MP imaging system (Bio-Rad, model: Universal Hood III)

7. Low-speed orbital shaker (Corning, model: 6780-FP)

8. Pierce power blotter (Thermo Scientific)

9. AccuBlock digital dry bath (Labnet International, model: D1100)

10. Tabletop centrifuge (Eppendorf, model: 5424)

11. Optima Max-XP tabletop ultracentrifuge (Beckman Coulter, Rotor TLA 100.3)

## Software and datasets

1. Fiji (ImageJ, free, no license needed, downloadable via link: https://imagej.net/downloads)

2. All data and code can be accessed in the supplementary materials of the original article [14] (https://doi.org/10.7554/eLife.90603.4, 10/31/2024)

3. Micro-Manager (downloadable via link: https://micro-manager.org/Download_Micro-Manager_Latest_Release)

## Procedure

**A. Transient transfection of one well of cells in a 6-well dish for cell imaging**

1. Equilibrate transfection reagent (FuGENE HD or Lipofectamine 2000) and Opti-MEM to room temperature (RT, ~25 °C). Warm DMEM + 10% FBS to 37 °C.

*Note: This transfection protocol can be performed using FuGENE HD or Lipofectamine 2000. DNA amounts have been optimized for each transfection reagent based on manual inspection of transfection efficiency across different DNA conditions.*

2. Ensure cells in one well of a 6-well dish are at 80%–90% confluency by manual inspection.

*Note: A subpopulation of cells will die from the toxicity of the transfection reagent, so it is better to plate at this higher density to ensure the majority of cell survival on the day of staining.*

3. Perform all the following steps in a tissue culture (TC) hood. Prepare HaloTag-Myo10-FlagTag plasmid stock at ~0.5 µg/µL and calmodulin plasmid stock at ~0.5 µg/µL. For FuGENE HD, proceed to step A4; for Lipofectamine 2000, proceed to step A5. Then, continue to step A6.

*Note: Co-transfection with calmodulin plasmid is not necessary, but could be useful. The Myo10-positive filopodia phenotype is not noticeably changed by the presence of additional calmodulin. See General notes for more details.*

4. If using FuGENE HD:

a. In a sterile 1.5 mL Eppendorf tube, prepare 1 µg of the HaloTag-Myo10-FlagTag plasmid and 0.2 µg of the calmodulin plasmid in a final volume of 200 µL of Opti-MEM. Pipette up and down 20× with a 200 µL tip, careful not to introduce air bubbles.

b. Add 3.5:1 FuGENE HD:DNA ratio (4.2 µL of FuGENE HD). Pipette up and down 20× with a 200 µL tip. Incubate the FuGENE/DNA mixture for 10 min at RT to allow complex formation, which will enable efficient cellular uptake of DNA.

5. If using Lipofectamine 2000:

a. In a sterile 1.5 mL Eppendorf tube, prepare 2.5 µg of the HaloTag-Myo10-FlagTag plasmid and 0.2 µg of the calmodulin plasmid in a final volume of 250 µL of Opti-MEM. Pipette up and down 20× with a 200 µL tip, careful not to introduce any air bubbles.

b. In another 1.5 mL Eppendorf tube, add 2.5:1 Lipofectamine 2000:DNA ratio (6.75 µL of Lipofectamine 2000) to Opti-MEM for a final volume of 250 µL. Pipette up and down 20× with a 200 µL tip.

c. Transfer the Lipofectamine solution to the DNA vial. Pipette up and down 20× with a 200 µL tip, careful not to introduce any air bubbles. Incubate the Lipofectamine/DNA mixture for 20 min at RT to allow complex formation, which will enable efficient cellular uptake of DNA.

6. Replace medium in well with 2 mL of DMEM + 10% FBS (warmed to 34–37 °C).

7. Add the total transfection reagent/DNA mixture (200 µL of the FuGENE/DNA mixture, or 500 µL Lipofectamine/DNA mixture) to one confluent well of cells, gently dispersing the mixture drop-by-drop from a 1,000 µL tip across the surface of the cell media. Gently shake the dish to disperse the mixture, then let cells grow in a TC incubator (37 °C, 5% CO_2_) for 24–48 h.

*Note: Cells grown at 48 h will have higher protein expression levels than at 24 h. For Myo10, we observed a doubled increase in protein expression with 48 h transfection vs. 24 h. Decide on a transfection time that is suited for your biological system.*

**B. Seeding, collecting cell pellets, and staining cells (fixed and live)**

1. Warm up PBS, DMEM + 10% FBS, and accutase to 37 °C.

2. Prepare 20 µg/mL laminin-coated coverslips in TC hood (see Recipe 1) right before seeding cells. Laminin promotes cell attachment to the glass surface, ensuring healthy cell spreading during staining.

a. For fixed cell experiments: 8-well chambered coverslip.

b. For live cell experiments: round coverslip AND 8-well chambered coverslip. Cells grown in the 8-well chambered coverslip will be fixed and used for measuring transfection efficiency.

3. After 24–48 h of transfection, aspirate the cell media from the well. Do a gentle 400 µL PBS wash, then add 400 µL of accutase to the well. Leave cells in the TC incubator for 15 min for accutase to detach the adherent cells.

*Note: After transfection, expect ~60%–80% cell viability.*

4. Add 500 µL of DMEM + 10% FBS. Collect cells in a 1.5 mL Eppendorf tube. Spin at 400× *g* for 3.5 min at RT.

5. Resuspend the pellet in DMEM + 10% FBS so that each well in the 8-well chambered coverslip and/or round coverslip is at ~50%–60% confluency and filled with at least 200 µL of media.

*Note: To achieve 50%–60% confluency, aim to seed 62,000–75,000 cells per well in the 8-well chambered coverslip and 100,000–300,000 cells on the round coverslip. Suspend cells in DMEM + 10% FBS at the desired concentration, then gently release cells into the corner of the well so as not to disturb the laminin coating. Ensure the cells are well-dispersed by manually moving the coverslip perpendicularly on the bench, avoiding rotational movement so cells do not clump in the center of the coverslip.*

6. Seed the unused cells into a well of the 6-well dish for SDS-PAGE analysis later. Return the 8-well chambered coverslip and/or round coverslip and 6-well dish to the incubator.

*Note: Cells are collected for SDS-PAGE shortly before staining for imaging to ensure that protein expression levels are comparable between SDS-PAGE analysis and microscopy experiments.*

7. After 2–3 h, cells should be attached. First, collect cells for SDS-PAGE analysis prior to fixing cells for microscopy. See section C.

8. Staining fixed cells in the 8-well chambered coverslip (for both live and fixed cell experiments):

a. Prepare the fixation and staining solution (see Recipes). Keep on ice.

b. Quickly aspirate media from all wells of the 8-well chambered coverslip. Quickly add 200 µL of cold fixation and staining solution to each well, releasing liquid at the corner of the wells so as not to dislodge the cells. Cover with foil and leave in the dark, undisturbed, at RT for 20 min.

c. Wash 3× with 250 µL of PBS (4 min each wash) and stain actin in cells with 200 µL of 1 mM Phalloidin-AF647 in 1% BSA (see Recipes). Incubate in the dark at RT for 20 min.

d. Wash 3× with 250 µL of PBS (4 min each) before immediate imaging.

e. If the fixed cell 8-well chambered coverslip sample is prepared for measuring transfection efficiency of live cell experiments, image this fixed sample after the live cell microscopy session.

*Note: To prevent photobleaching, cells should be kept in the dark when not being imaged. Image within hours of preparation to ensure samples are fully labeled and not degraded.*

9. Staining live cells on the round coverslips:

a. Label cells on round coverslips with 200 µL of DMEM + 10% FBS containing 0.75 µM TMR-HaloLigand. Leave in the TC incubator for 15 min.

b. Gently wash 2× with 200 µL of DMEM + 10% FBS (warmed to 34–37 °C).

c. Image the sample in 200 µL of DMEM + 10% FBS containing 1× GOC.

**C. Collecting cell lysates for SDS-PAGE**

1. Warm up PBS, DMEM + 10% FBS, and accutase to 37 °C. Perform all the following steps in a TC hood at the same time as cells are ready to be stained for microscopy.

2. Aspirate cell media from the transfected well in the 6-well dish. Do a gentle 400 µL PBS wash, then add 400 µL of accutase. Leave cells in the TC incubator for 15 min.

*Critical: For live-cell experiments, the accutase should also contain 0.75 µM TMR-HaloLigand. Labeling cells while they are still alive, prior to pellet collection, more accurately reflects HaloTag labeling accessibility during live microscopy.*

3. Add 500 µL of DMEM + 10% FBS to the well. Collect cells in a 1.5 mL Eppendorf tube. Spin at 400× *g* for 3.5 min at RT.

4. Resuspend the pellet in 200 µL of DMEM + 10% FBS; note that this volume will depend on how many cells you have. Remove 10 µL of cells and combine with 10 µL of 0.4% Trypan blue to count using a hemocytometer. Add another 800 µL of DMEM + 10% FBS to the vial of remaining cells and spin at 400× *g* for 5 min at RT.

5. Remove the supernatant with a pipette, leaving only the cell pellet in the tube. Use the cell pellet for immediate processing or move the tube directly to -80 °C for storage. Process frozen cell pellets within a year.

**D. Converting Myo10 signal from SDS gel and microscopy to molecules**

1. Prepare cold RIPA buffer (see Recipes) with 1× protease cocktail inhibitor and 1 mM PMSF. Keep on ice.

2. Calculate how much RIPA buffer to suspend the cell pellet so that 5 µL of cell suspension corresponds to 50,000 cells. Pipette each cell pellet 20×, making sure not to generate air bubbles. Leave on ice for 15 min.

3. Spin at 15k × *g* for 10 min at 4 °C. Pipette the supernatant to a new tube without touching the pellet.

4. Incubate 5 µL of lysate from the fixed cell experiments (50,000 cells/5 µL) with 2 µM TMR-HaloLigand in a 10 µL total volume. Leave on ice for 10 min.

*Note: Any high concentration of TMR-HaloLigand is acceptable (e.g., 2 µM), provided it is in excess relative to Myo10 to ensure complete labeling of tagged-Myo10. Excess TMR-HaloLigand will run faster than the cell lysate during SDS-PAGE. It is unnecessary to incubate the lysates from the live cell experiments because they are already labeled with TMR-HaloLigand.*

5. Prepare a 1:500 dilution of the HaloTag standard protein with 0.5 µM TMR-HaloLigand in PBS. Using the 500× dilution, aliquot in protein LoBind tubes at 1.25, 2.5, 5, 10, and 15 ng in a 10 µL total volume of PBS.

*Note: If the stock solution of HaloTag standard protein is at 3 mg/mL, then a 1:500 dilution yields a 6 µg/mL solution. Refer to the manufacturer's product sheet for the lot-specific starting concentration of HaloTag standard protein, since it varies by batch.*

6. Add 2.5 µL of 5× SDS loading buffer to all the cell lysates and HaloTag standard protein samples.

7. Heat protein samples at 70 °C for 10 min before loading 12.5 µL onto a 4%–20% Mini-PROTEAN TGX stain-free protein gel. Run gel at 180 V for 45 min.

8. Take images of the gel on a ChemiDoc in the stain-free, AF647, and rhodamine channels.

*Note: The stain-free channel (standard filter, UV trans illumination) gives total protein signal, the AF647 channel (695/55 filter, red epi illumination) visualizes the protein ladder, and the rhodamine channel (605/50 filter, green epi illumination) shows TMR.*

**Figure 1.**
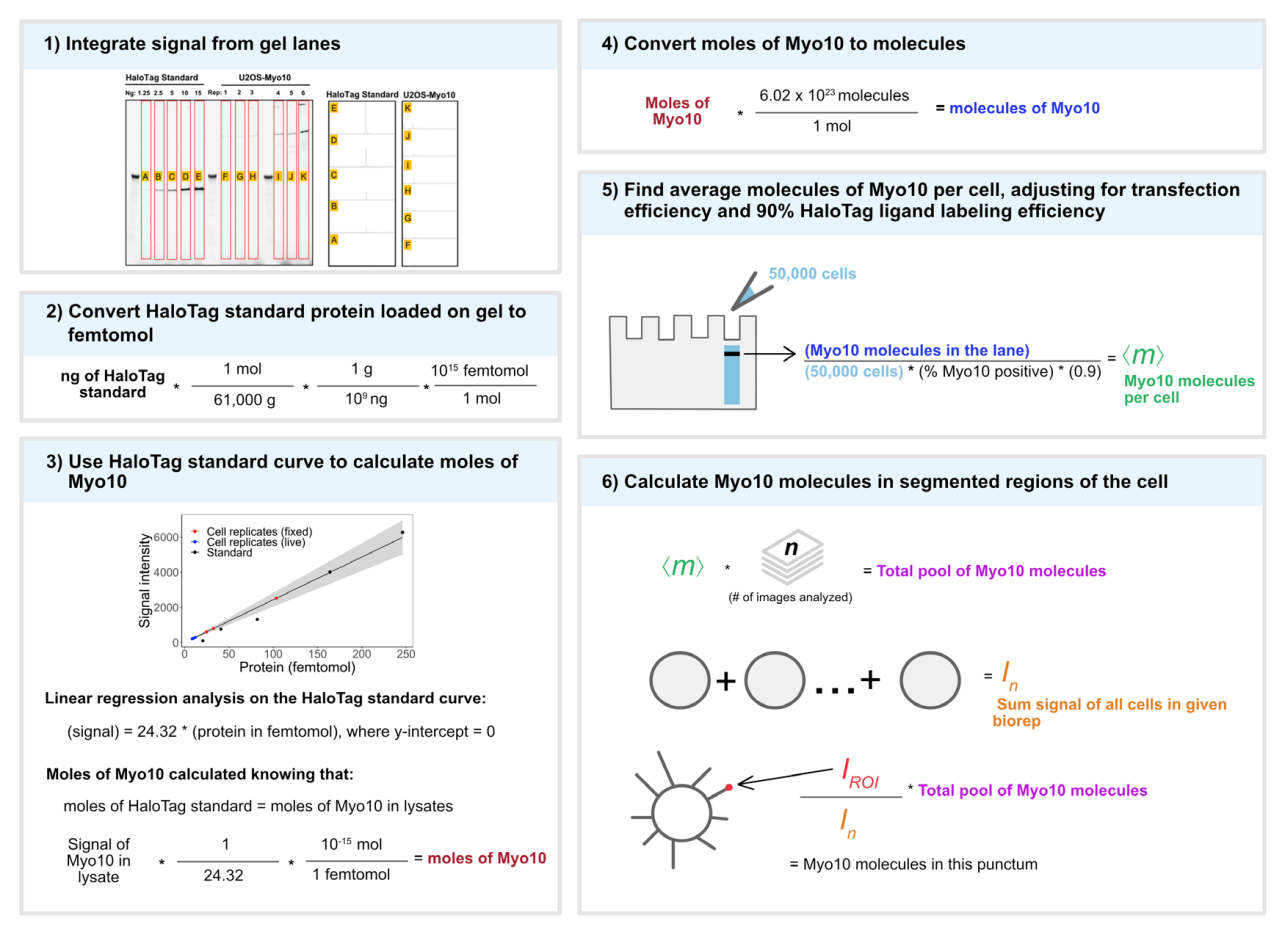
Conversion of Myo10 signal from SDS-PAGE and microscopy to molecule counts. 1) TMR illumination of the SDS-PAGE gel. HaloTag standard protein (lanes A–E, loaded in ng stated above each lane) and cell lysates from six separate U2OS transient transfections (50,000 cells except lane J has 10,000 cells). Lanes F, G, and H: for live-cell analysis. Lanes I, J, and K: for fixed-cell analysis. Bands in the unlabeled wells are from the protein ladder. Signal was integrated for full-length Myo10 (at ~250 kDa) and aggregation in the wells using ImageJ’s Gel Analysis plug-in. 2) The ng amount of HaloTag standard protein loaded is converted to femtomol. 3) Standard curve for TMR signal of HaloTag standard protein (black dots) vs. signal from HaloTag-Myo10 U2OS. y = 24.32x, where the y-intercept is set to 0; R^2^ = 0.98. Red dots = fixed-cell experiments; blue dots = live-cell experiments. Gray shading = 95% confidence interval. 4) Converting moles of Myo10 in cells to molecules. 5) Finding the average molecules of Myo10 per cell. 6) Relating SDS-PAGE and microscopy signal to Myo10 molecule counts in segmented regions of the cell. Adapted from [14].

9. Use the Gel Analysis plug-in in ImageJ to measure signal intensity of the gel bands (Figure 1).

a. Use the rectangle tool to draw a box around the first gel band. Include ample space on either side of the gel band when drawing the rectangle for signal integration. Then navigate to *Analyze* → *Gels* → *Select First Lane*.

b. Box the subsequent gel band: *Analyze* → *Gels* → *Select Next Lane.* Do this for all gel bands.

c. Once all the gel bands are boxed, generate the lane profile plots: *Analyze* → *Gels* → *Plot Lanes.*

d. Integrate the signal under the peak. First, use the straight-line selection tool to draw baselines for each peak so that it creates a closed shape. Second, measure the area under each peak by using the wand (tracing) tool and clicking inside the enclosed shape.

10. Plot a standard curve of the HaloTag standard protein TMR signal to estimate the number of Myo10 molecules per cells loaded. Adjust the molecule count by the transfection rate obtained from microscopy and a 90% HaloTag ligand labeling efficiency (Figure 1).

**E. Calculate the number of Myo10 molecules per cell from the SDS gel**

The R code to run a Myo10 signal-to-molecules calibration can be found in the section “Additional files” of the original article [14] in the link “Source code 1.” Open folder *MoleculeCalibration* → *SignaltoMolecules.Rmd*. Plug in the signal values integrated from the SDS gel lanes of each cell lysate bioreplicate and HaloTag standard protein dilution series. The code runs a linear regression analysis on the HaloTag standard protein curve for a Myo10 signal-to-molecules conversion. It provides the lower and upper bounds of a 95% confidence interval.

**F. Myo10 western blot**

1. Combine 5 µL of cell lysate (at 50,000 cells/5 µL; WT and Myo10-transfected samples) with 5 µL of PBS and 2.5 µL of 5× SDS loading buffer.

2. Heat protein samples at 70 °C for 10 min before loading 12.5 µL onto a 4%–20% Mini-PROTEAN TGX stain-free protein gel. Run gel at 180 V for 45 min.

3. Take images of the gel on a ChemiDoc in the stain-free and AF647 channels to visualize total protein signal and ladder. Place the gel into 1× semi-dry transfer buffer.

4. Prepare 1 L of 1× TBST: 100 mL of 10× TBS, 1 mL of 0.1% Tween 20, and 899 mL of H_2_O.

5. Soak filter papers in 1× semi-dry transfer buffer (see Recipes).

6. Activate the PVDF membrane by submerging it in methanol.

7. Assemble blot “sandwich” in the following order (from bottom to top):

a. Two filter papers

b. Membrane

c. Gel (cut to the dimensions of filter papers)

d. Two filter papers

**Figure 2.**
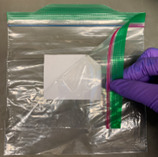
Developing western blot for visualization. Incubate the blot in an equal mix of peroxide and enhancer inside a Ziploc for 2 min before imaging on a ChemiDoc.

**Figure 3.**
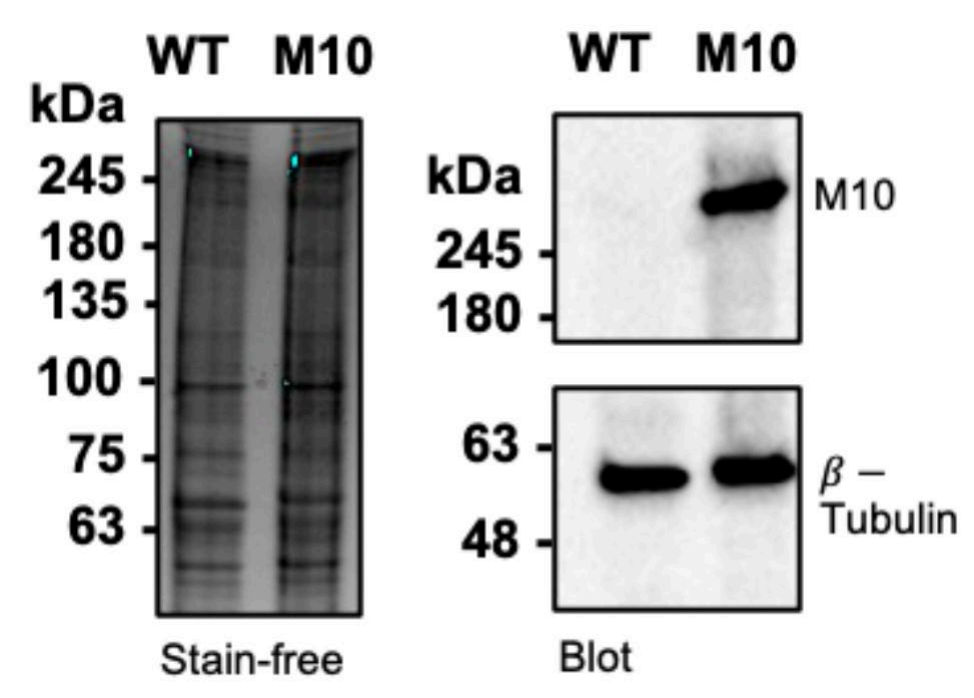
Western blot of Myo10 in U2OS cells. Left: SDS-PAGE gel of wildtype U2OS (WT) vs. U2OS transfected with HaloTag-Myo10 (M10). 50,000 cells per lane. Right: Western blot of Myo10 (NBP1-87748) and β-tubulin and loading control (Invitrogen, catalog number: 22833). There are very low endogenous Myo10 levels in U2OS. Image is reproduced from [14].

8. Roll out air bubbles, then pour 2–3 mL of 1× semi-dry transfer buffer on top to keep it wet.

9. Run blot at 1.3 A constant current and 25 V for 12 min.

10. Block membrane with 5% milk in 1× TBST for 1 h at RT, shaking at 60 rpm.

11. Stain membrane with primary antibodies (10,000× dilution anti-myosin10 rabbit polyclonal, 10,000× dilution anti-beta tubulin mouse monoclonal) in 5% milk in 1× TBST. Leave shaking overnight at 4 °C, or at RT for 1 h, at 60 rpm.

12. Wash 3× with 5% milk or 1× TBST (10 min each wash, shaking at 60 rpm).

13. Stain membrane with secondary antibodies (10,000× dilution rabbit HRP, 10,000× dilution mouse HRP) in 1× TBST. Shake at RT for 1 h at 60 rpm.

14. Wash 3× with 1× TBST (10 min each wash, shaking at 60 rpm).

15. Right before imaging the blot, mix 1 mL of SuperSignal West Femto stable peroxide with 1 mL of Super West Femto luminol/enhancer, then incubate the blot for 2 min inside a Ziploc (Figure 2). Handle the blot with tweezers.

16. Take images of the gel on a ChemiDoc with the setting “Chemi Hi Resolution” (Figure 3).

17. If the number of Myo10 molecules in the transfected cell lysate is known (Figure 1), the ratio of western blot signals between the two lanes can be used to estimate what proportion of the Myo10 signal in the transfected sample comes from endogenous protein (Figure 3).

**G. Verifying labeling efficiency of TMR-HaloLigand**

1. Dilute the HaloTag standard protein to 8 µM in a 20 µL reaction volume of PBS. Add TMR-HaloLigand in 5× molar excess (final 40 µM). Incubate on ice for 10 min.

*Note: For reference, the HaloTag standard protein is 61,000 g/mol. HaloTag standard protein provided at 3 mg/mL is 49.18 µM. To prepare HaloTag standard protein at 8 µM, combine 3.25 µL of HaloTag standard protein with 1.6 µL of TMR-HaloLigand (500 µM) and 15.15 µL of PBS. 500 µM TMR-HaloLigand can be prepared by combining 1 µL of stock 5 mM TMR-HaloLigand with 9 µL of DMSO.*

2. Measure the A_280_ with a NanoDrop spectrophotometer as the “pre-beads” concentration; this is to verify that the starting concentration is as expected. In the NanoDrop menu, select *Proteins* → *Protein A_280_* → *Sample type: 1 Abs = 1 mg/mL (baseline correction at 340 nm)*.

3. Fill a 1.5 mL protein LoBind tube with Bio-Beads SM-2 to the ~10 µL volume mark. Wash beads by vortexing and discarding each wash buffer as follows: 400 µL of methanol 3×, distilled water 3×, and PBS 3×. Vortex for 3 s and pipette off buffer after each wash.

4. Aspirate liquid and add 20 µL of labeled HaloTag standard protein. Incubate for 2 h at RT in the dark.

5. Spin down the beads at 600× *g* for 3 min at RT.

**Figure 4.**
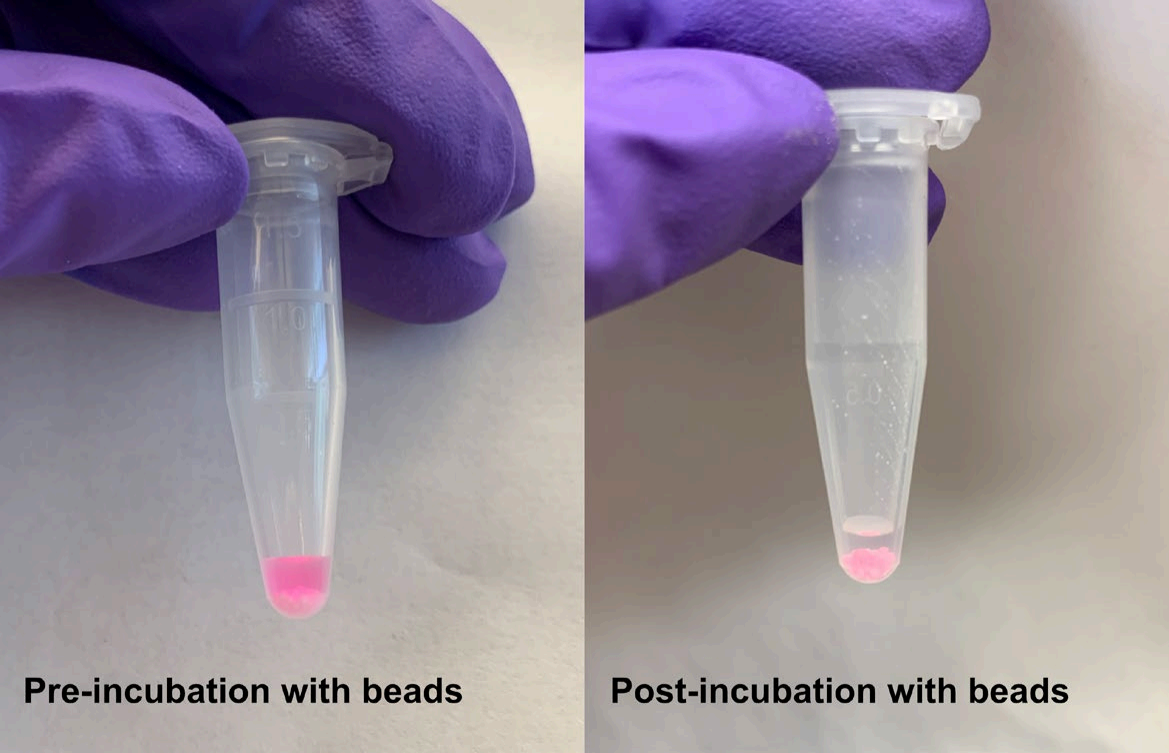
Absorbing excess TMR-HaloLigand with Bio-Beads. Left: Protein solution is pink when excess TMR-HaloLigand is unbound. Right: Protein solution becomes clear once excess TMR-HaloLigand is bound by Bio-Beads.

*Note: At this point, the solution should no longer be pink because excess TMR-HaloLigand should be absorbed by the beads (Figure 4). If the beads are not pink and the solution is still pink, use more beads or let the incubation proceed longer.*

**Figure 5.**
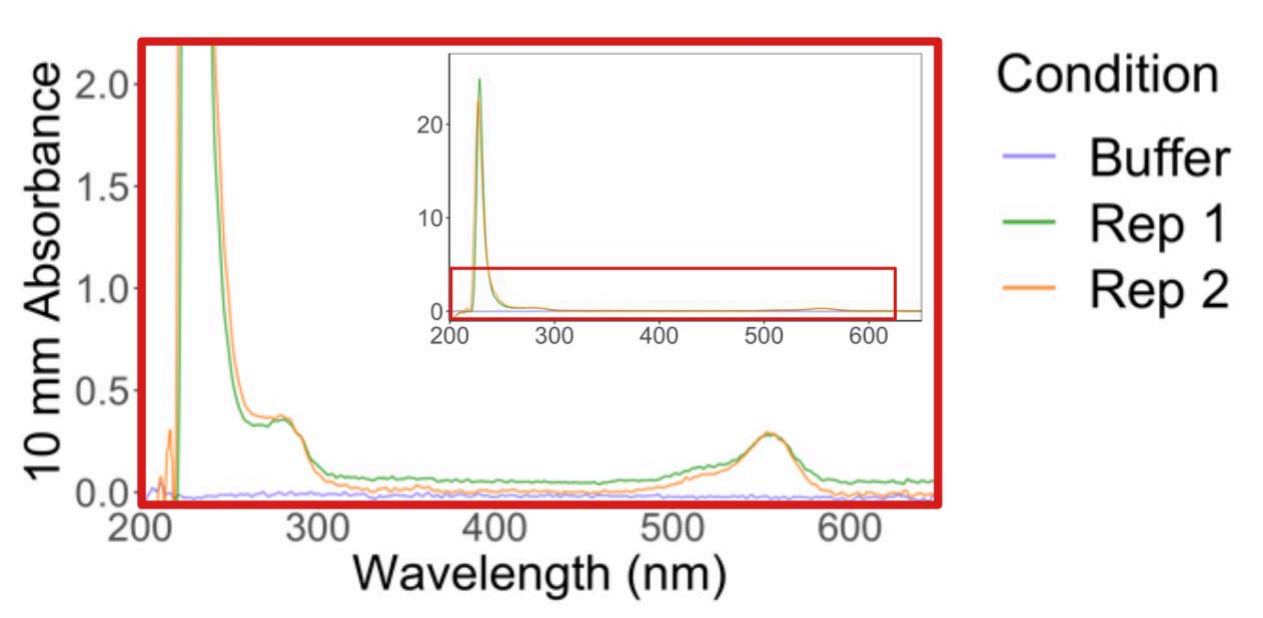
Absorbance spectrum of post-bead labeled HaloTag standard protein. Spectrum shown is a zoom-in of the red boxed region in the top right inset. Plot is adapted from [14].

6. Recover the supernatant and measure the protein and dye absorbances (Figure 5): in the NanoDrop menu, select *Proteins* → *Protein A280* → *Sample type: 1 Abs = 1 mg/mL (baseline correction at 340 nm).* Measure absorbance at 533 nm: in the NanoDrop menu, select *Custom* → *UV-Vis* → *533 nm.*

Note: For reference, the TMR extinction coefficient is 7.8 × 10^4^ M^-1^·cm^-1^. To calculate the concentration of TMR-HaloLigand from absorbance, use the formula: concentration = absorbance/(extinction coefficient * path length). See Table 1 for example calculations.

7. Combine 10 µL of protein and 2.5 µL of 5× SDS loading buffer. Heat protein samples at 70 °C for 10 min before loading 12.5 µL onto a 4%–20% Mini-PROTEAN TGX stain-free protein gel. Run gel at 180 V for 45 min.

8. Take images of the gel on a ChemiDoc in the stain-free and rhodamine channels.

9. Use the Gel Analysis plug-in in ImageJ to measure signal intensity of the gel bands.

*Note: Knowing this value allows you to account for protein that may have nonspecifically bound to the beads. Compare the signal intensities of pre- and post-bead gel bands to correct for the post-beads protein concentration value. See Table 1 for example calculations.*

**Table 1.** Calculating HaloTag degree of labeling with TMR-HaloLigand. Example calculations for two technical replicates. (A) Starting concentration of HaloTag standard protein. (B) Ratio of the integrated signal of HaloTag standard protein well from gel, pre-, and post-beads. (C) Multiply (A) by (B) for the concentration of HaloTag standard protein post-beads, which is also the expected concentration of TMR-HaloLigand if 100% labeling has occurred. (D) Absorbance at 553 nm of protein solution post-beads. (E) Concentration of TMR-HaloLigand based on (D). (F) Percentage of HaloTag standard protein labeled by TMR-HaloLigand, calculated by (E)/(C). Table is adapted from [14].

Measurement	Rep 1	Rep 2
(A) Pre-beads protein (µM)	6.8	6.8
(B) Post/pre-beads gel signal	0.58	0.62
(C) Post-beads expected dye (µM) (i.e., post-beads protein)	3.9	4.2
(D) Post-beads Abs553	0.28	0.30
(E) Post-beads dye (µM)	3.6	3.8
(F) % dye labeling	0.91	0.90

**H. Ensuring TMR-HaloLigand labeling saturation in cells (fixed and live)**

1. Seed cells onto laminin-coated coverslip as described in section B.

a. For fixed cells: 6 wells of an 8-well chambered coverslip.

b. For live cells: 6 round coverslips.

**Figure 6.**
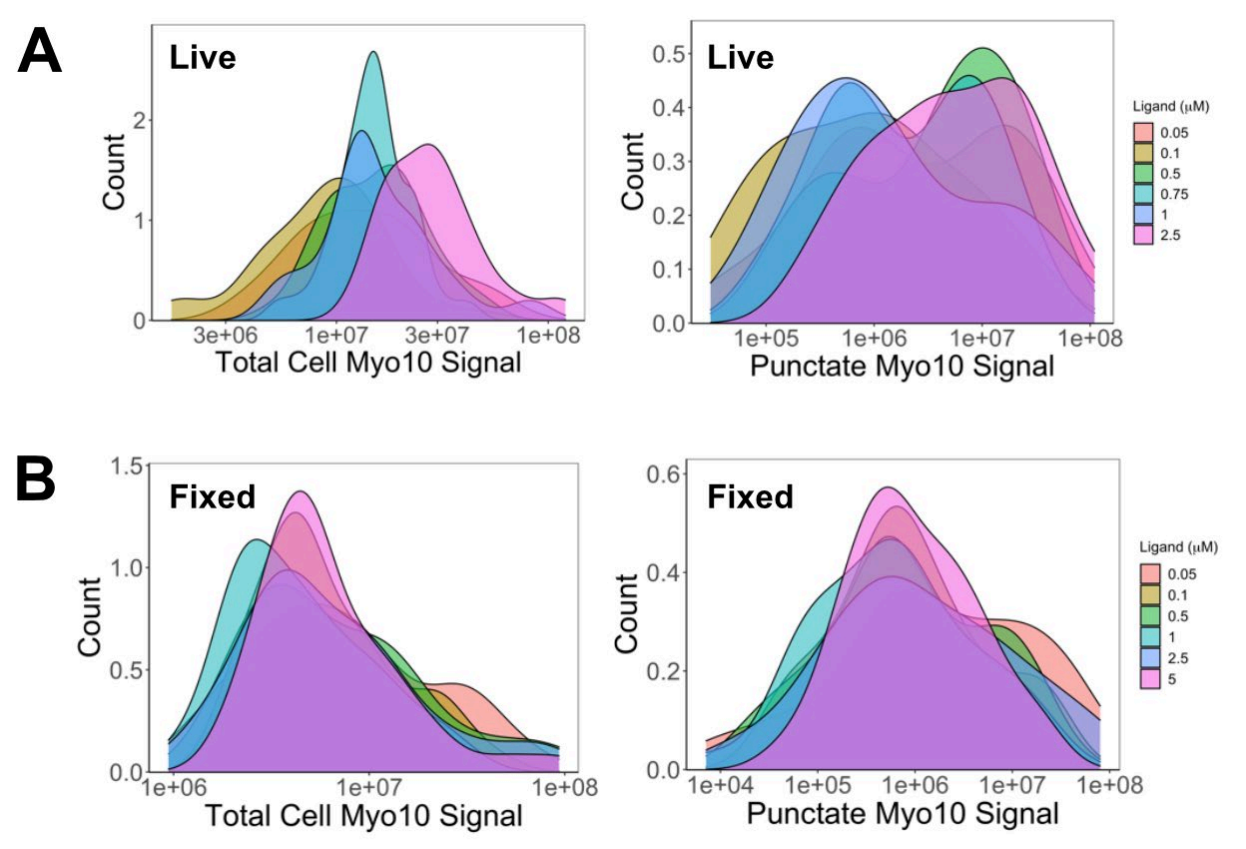
0.5 µM TMR-HaloLigand saturates Myo10 labeling in U2OS cells. (A) In live cells: Higher HaloTag ligand-TMR concentrations increased total intracellular Myo10 signal (left) due to uneven background, artificially inflating measurements. Punctate signal (right) reveals true saturation at 0.5 µM. (B) In fixed cells: Total (left) and punctate (right) Myo10 signals saturate at 0.5 µM TMR-HaloTag ligand. Based on Table 1, we estimate that 90% of HaloTag-Myo10 is labeled at 0.5 µM saturation in both live and fixed cells. Image is reproduced from [14].

2. Create a 20 µM dilution of TMR-HaloLigand into DMSO. In the following steps, working solutions will be made at the final concentrations of 0.05, 0.1, 0.5, 0.75, 1, and 2.5µM.

3. Staining fixed cells:

a. Make a fixation buffer base containing 4% PFA, 0.08% Triton, and 1:1,000 DAPI in PEM buffer. Keep on ice.

b. Add TMR-HaloLigand to reach the final concentrations listed in step H2. Keep on ice.

c. Quickly aspirate cell media from all 6 wells and quickly add 200 µL of the fixation/staining buffer to each well, releasing liquid at the corner of the wells so as not to dislodge the cells. Leave in the dark, undisturbed at RT for 20 min.

d. Wash 3× with 250 µL of PBS (4 min each wash) and stain actin in cells with 200 µL of 1 mM Phalloidin-AF647 in 1% BSA (see Recipes). Incubate in the dark at RT for 20 min.

e. Wash 3× with 250 µL of PBS (4 min each) before immediate imaging (Figure 6A).

3. Staining live cells:

a. Add TMR-HaloLigand into prewarmed (34–37 °C) DMEM + 10% FBS to prepare 200 µL solutions at the final concentrations listed above. Incubate in the dark in the TC incubator.

b. Gently wash 2× with 200 µL of DMEM + 10% FBS (warmed to 34–37 °C).

c. Image the sample in 200 µL of DMEM + 10% FBS containing 1× GOC (Figure 6B).

**I. Imaging cells (fixed and live)**

1. Use an Axiovert microscope with a 60× water objective (or any other suitable objective). The signal read-out should be below detector saturation.

a. For live cells only: Heat the objective to 37 °C for 45 min prior to imaging to equilibrate the temperature of the objective.

*Note: A water objective is not crucial. Choose an objective with a magnification sufficient to capture the whole cell (>60×) and a high numerical aperture (>1.2×) to resolve fine structures like filopodia.*

2. Imaging fixed cells:

a. Set camera gain (if using) and exposure. Pixel values should use the entire dynamic range of the camera, but must be below camera saturation.

b. Image TMR channel first (520 nm green light) and then AF633 (630 nm red light). Image immediately upon illumination of the field of view. Save the image as a stack in Micro-Manager.

c. Using the same camera exposure and gain settings, collect 50 images for each bioreplicate, collecting 150 images across three bioreplicates.

3. Imaging live cells:

a. Set camera gain (if using) and exposure; 300 ms is a recommended exposure time since it captures sufficient signal of the entire filopodial lifecycle. Pixel values should use the entire dynamic range of the camera, but must be below camera saturation.

b. Record 10 random cells in the TMR channel, recording videos for <6 min or until the cell starts dying and/or rounds up. Save each movie as a stack in Micro-Manager.

c. Using the same camera exposure and gain settings, collect 30 images across 3 bioreplicates. For each bioreplicate, take snapshots of ~50 living cells to sample the fluorescence intensity distribution for the signal-to-molecule conversions.

4. Record the transfection efficiency using the fixed-cell sample. Survey at least 100 random cells to check the transfection rate. Scan across the sample, stopping to image whenever cells are in frame. First, count the number of cells with DAPI signal in the UV channel, then change to the TMR channel to count the number of Myo10 positively expressing cells. Record this transfection rate. It is not necessary to store these images.

## Data analysis

All code for the subsequent data analysis sections can be found in the section “Additional files” of the original article [14] in the link “Source code 1.”

Scripts were run on a macOS 14.5, platform x86_64-apple-darwin17.0 (64-bit). Table 2 contains information about the libraries used in the Python and R scripts.

**Table 2.** Versions of the R and Python libraries used in the source code

R version 4.1.2 (2021-11-01)	Python 3.9.5
readr_2.1.3	scipy==1.7.2
stringr_1.5.0	Pillow==8.4.0
dplyr_1.0.10	pandas==1.4.3
ggplot2_3.3.6	numpy==1.26.4
plotly_4.10.0	napari==0.4.16
tidyr_1.2.1	matplotlib==3.5.0
scales_1.2.1	
purrr_0.3.5	
ggpmisc_0.5.1	
patchwork_1.1.2	
usefr_0.1.0	
rmarkdown_2.17	

**A. Analyzing fixed-cell images**

The Python scripts used to process and analyze the fixed cell data can be found in the folder *Cell Segmentation*. Example actin and Myo10 TIFF files are provided.

**Figure 7.**
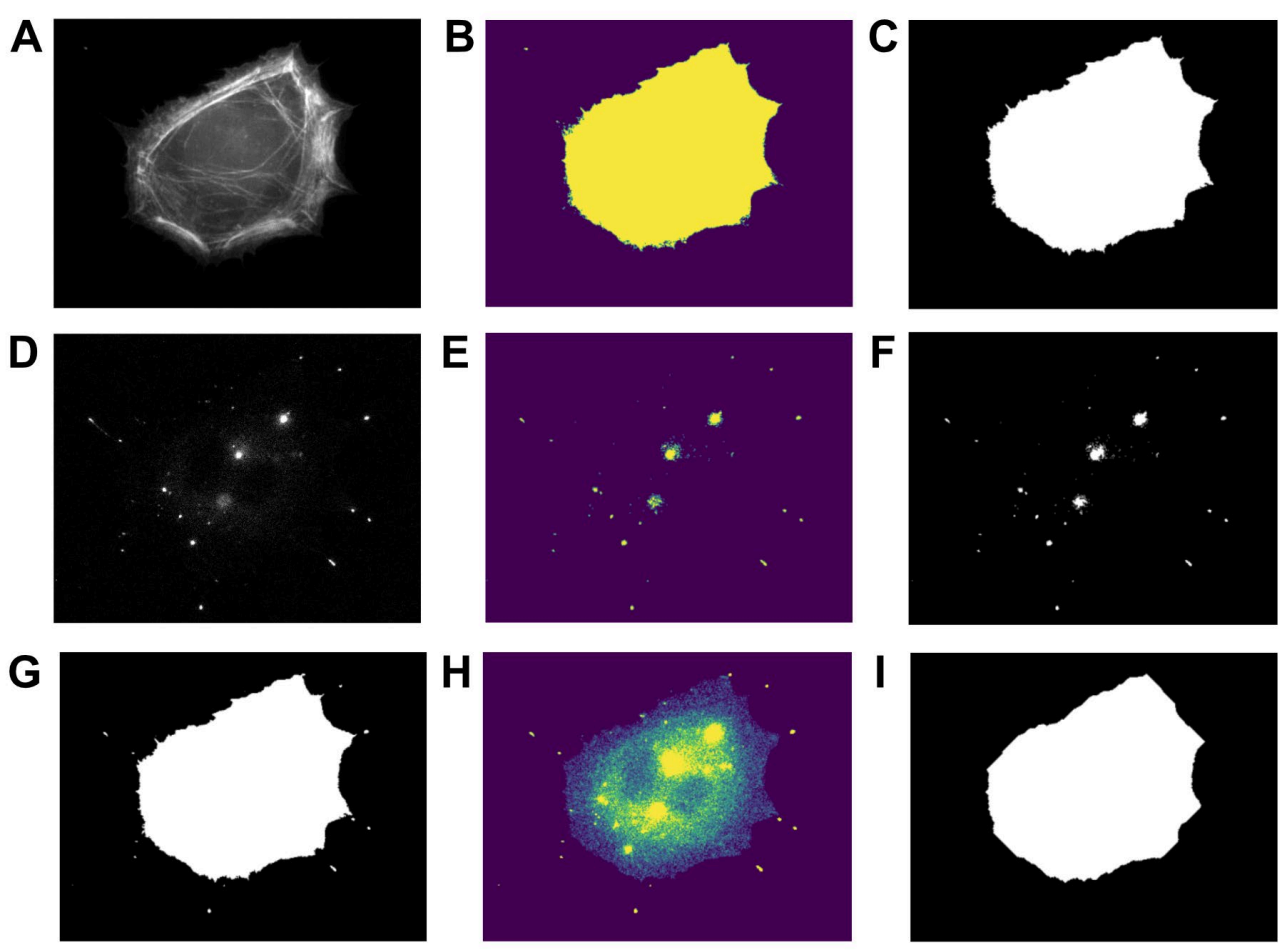
Output of cell body vs. filopodia segmentation image analysis. (A) Phalloidin-stained cell image. (B) Actin mask after watershed segmentation. (C) Final actin mask after filling holes in the mask from (B) and filtering out non-cell objects. (D) Myo10-stained image. (E) Myo10 mask after watershed segmentation. (F) Final Myo10 mask after filling holes in the mask from (E). (G) Total cell mask. (H) Signal of the Myo10-stained image, restricted to the region defined by the “total cell mask.” (I) Cell body mask.

1. Find cell body vs. filopodial Myo10 signal

a. Using “CellSegmentation_Manuscript.ipynb,” set the correct file path to the raw tiff file of the phalloidin-stained cell image (Figure 7A).

b. Filter the phalloidin-stained cell image to remove non-cell objects and apply watershed segmentation (see Python functions used below). This generates a mask of the actin area (Figure 7B, C).

# Separate actin signal from background

Amarkercutoff = 2000 # Manually set cut-off value

Amarkers = np.zeros_like(Agrayarr)

Amarkers[Agrayarr < Amarkercutoff] = 1 # background

Amarkers[Agrayarr > Amarkercutoff] = 2 # object

from skimage.segmentation import watershed

# Watershed segmentation

Asegmentation = watershed(Aelevation_map, Amarkers)

# Filling holes in binary mask sometimes improves mask

Asegmentation = ndi.binary_fill_holes(Asegmentation - 1)

# Filter out non-cell objects, if any

Aobjectcutoff = 100 # Manually set cut-off value

Alabel_objects, Anb_labels = ndi.label(Asegmentation)

Asizes = np.bincount(Alabel_objects.ravel())

Amask_sizes = Asizes > Aobjectcutoff

Amask_sizes[0] = 0

Asegmentation2 = Amask_sizes[Alabel_objects] # Final actin mask

c. Set the correct file path to the raw tiff file of Myo10-stained cell image (Figure 7D).

d. Filter the Myo10-stained image to remove non-cell objects and apply watershed segmentation; use the same Python functions as for the phalloidin-stained image. This generates a mask of the Myo10 area (Figure 7E, F).

e. Combine the actin and Myo10 masks to obtain the “total cell mask” (Figure 7G).

f. Subtract background from the Myo10 TIFF image. Use this background-subtracted array to calculate the total Myo10 cell signal in the “total cell mask” (Figure 7H).

*Note: To find an average background value of the Myo10 TIFF image, you can open the Myo10-stained image in Fiji. Draw a square on the background (e.g., 56 × 56 pixels) near the cell body. Calculate the average signal of the square, then subtract this value from each pixel value of the Myo10 TIFF image array.*

g. Generate a “cell body mask” from the phalloidin-stained cell image (Figure 7I). Adjust the parameters of the erosion function and opening function as desired (see Python functions used below). Use the background-subtracted Myo10 array to calculate the total Myo10 cell signal in the “cell body mask.”

from scipy import ndimage as ndi

# Erosion function on actin mask; manually set iterations as appropriate

body_cell = ndi.binary_erosion(Asegmentation2, iterations = 1)

plt.imshow(body_cell,cmap='gray')

# Opening function on actin mask; manually set iterations as appropriate

body_cell2 = ndi.binary_opening(body_cell, iterations = 20)

plt.imshow(body_cell2,cmap='gray')

h. Subtract the “cell body mask” from the “total cell mask” for a “filopodia mask.” This gives the total filopodial Myo10 signal.

**Figure 8.**
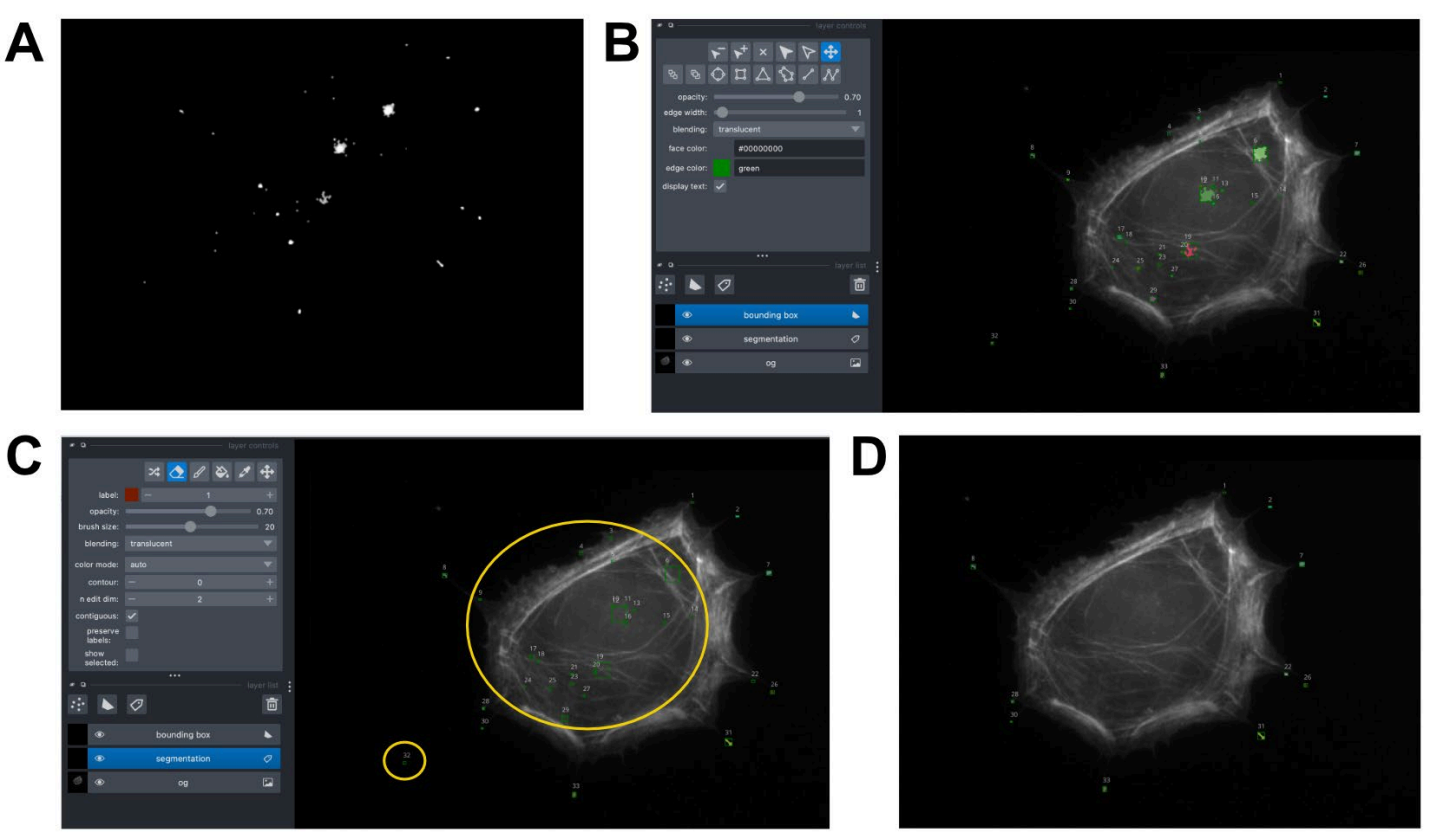
Output of filopodial puncta segmentation image analysis. (A) Signal of the Myo10-stained image, restricted to the region defined by the mask shown in Figure 7F. (B) Myo10 filopodial puncta segmented by Connected Component Analysis, visualized in Napari. Each segmented punctum is color-coded and has a labeled bounding box. The phalloidin-stained image is included in the background as a reference for where filopodial tips are. (C) Non-filopodial puncta (contained in the yellow circles) can be removed with the eraser tool in the “segmentation” layer of Napari. (D) Final segmented filopodial puncta for analysis.

2. Find individual Myo10 filopodial puncta signal

a. Using “FilopodialPunctaSegmentation_Manuscript.ipynb,” set the correct file paths to the raw tiff files of the phalloidin-stained and Myo10-stained cell images (Figure 7A, D).

b. Filter the Myo10-stained image to remove non-cell objects and apply watershed segmentation. This generates a mask of the Myo10 area. Find the total Myo10 signal in the mask (Figure 8A).

*Note: You can have relaxed parameters during this filtering step because segmented puncta that do not belong to filopodia can be manually erased later.*

c. Subtract the background value from each pixel of the Myo10 TIFF image for a background-subtracted Myo10 signal array.

d. Perform connected component analysis (CCA) to create masks for all filopodia-localized Myo10 puncta.

*Note: Function used: skimage.measure.label from the scikit-image library. In the CCA, pixels are considered neighbors if they are linked by at most two orthogonal jumps. A detailed explanation can be found in the original documentation [23].*

e. Run Napari [24]. Run the code block titled “MANUALLY ERASE UNDESIRED SPOTS” and inspect the segmented puncta (Figure 8B). Selecting the “segmentation” layer, manually erase noise, false segmentations, and cell body signal with the eraser tool. Close out of the Napari window and re-run the same code block to view the updated ROIs (Figure 8C). Continue doing this until you are satisfied (Figure 8D). Then run the next cell to see the signal of each punctum. Save.

*Note: It is recommended to screenshot the final Napari window with the correct final segmentations because it is useful to see the label of each puncta segmentation for downstream processing.*

f. Open the saved file containing the labeled puncta signal in Excel. Check the ROI labels with the segmented cell image. Manually combine puncta that were segmented separately but are actually part of the same filopodium mask.

**B. Convert fluorescence intensities into the number of Myo10 molecules**

In Figure 1, panel 6, I_n_ represents the sum of the fluorescence signal for n = 50 background-subtracted cell images. This fluorescence signal comes from nm molecules, where m = expected number of Myo10 molecules per transfected cell (from SDS-PAGE analysis). I_ROI_ represents the background-subtracted and summed signal of a single region-of-interest (e.g., a filopodial punctum). The number of Myo10 molecules in a region of interest, r, is r = nm * I_ROI_/I_n_. Perform the calculations with the m, n, and I_n_ values for each bioreplicate.

**C. Orientation of Myo10 distribution in fixed-cell filopodia**

The R code to plot distributions can be found in the folder *Roseplots* → *Myo10_Roseplots.Rmd*. The original data from [14] are contained in the files “HighSignalNorth_MeanMolecules.csv” and “LowSignalNorth_MeanMolecules.csv.”

1. Find the cell’s center of mass and divide it into 20 radial sections from the cell’s center.

2. Average the Myo10 filopodial molecules within each section.

3. Align the section with the highest molecules to degree 0 for each cell’s rose plot.

*Note: It is easier to count puncta that are at the border of two radial sections once for each section.*

4. Alternatively, you can also generate a rose plot displaying regions of low/no Myo10 surrounded by low signal.

*Note: If more than one section contains no Myo10, randomly select an empty Myo10 section to align to degree 0.*

**D. Local concentration of Myo10 in fixed-cell filopodia**

The R code to plot the Myo10 concentrations in filopodial tips can be found in *FilopodialTipConcentrations* → *TipDensity.qmd*. The original data from [14] is in the file “TipConcentration.csv.”

1. Open the Myo10-stained image in Fiji. Randomly select Myo10 tip-localized puncta from each bioreplicate set for analysis.

2. With the straight-line tool, measure the length of each punctum and record the value in an Excel spreadsheet. Include the punctum’s signal, which is the value obtained from step A2 (Data analysis).

3. Estimate the volume occupied by the Myo10 punctum using the volume of a cylinder: length = height and width = 2*radius, where radius is assigned as 100 nm (reported average filopodial radius [25]) due to the resolution limit. Convert signal intensity of the Myo10 punctum to molecules as described earlier. Calculate molarity, where molarity = mol/cm^3^.

4. In the R script, set the file path to your csv file containing columns of radius, length, volume, signal, molecules, and molarity. Plot.

**E. Tracking molecules and velocity of Myo10 puncta in live cells**

The R script for velocity analysis can be found in the folder *LiveCellAnalysis* → *Fig4_VelocityAnalysis.Rmd*. The original data from [14] are contained in the *Set1, Set2*, and *Set3* subfolders.

To get a live cell intensity distribution for the fluorescence intensity-to-molecule conversions, manually draw the bounding edges of the cells using the filopodial puncta as a guide in Fiji. Use this hand-drawn mask to measure the total intracellular Myo10 signal per cell. Convert the fluorescence signal intensity to the number of molecules as described in section B of Data analysis.

1. Open the Myo10 live cell movie in Fiji and subtract the background using a rolling ball radius of 50 pixels: *Process* → *Subtract Background…*

2. Initiate the Fiji plug-in TrackMate [26]. Use the following parameters:

a. LoG detector with estimated blob diameter = 1 µm with median filter and sub-pixel localization selected.

b. Set filters (e.g., quality) on spots as desired.

c. LAP tracker with frame to framelinking max distance = 0.5 µm, track segment gap closing max distance = 1 µm (max frame gap = 2); track segment splitting max distance = 0.5 µm, track segment merging max distance = 1 µm.

d. Set filters on tracks as desired.

3. After TrackMate segmentation is complete, save the TrackMate project and export as csv the *All spots table*. In TrackMate, manually inspect all trajectories in the cell movies and record the track IDs for these three types of filopodial events in an Excel sheet:

a. Initiation: a Myo10 punctum gathers at the plasma membrane and initiates into a filopodium.

b. Second-phase elongation: a new Myo10 punctum grows from an existing one.

c. Retraction: a Myo10 punctum, detected in the movie’s first frame, moves toward the cell body.

*Note: Define the start of the event by when TrackMate first segments the punctum.*

**Critical:** Inspect each movie in TrackMate to ensure the trajectories are accurate.

*Note: Exclude analysis of segmented puncta that are clearly background noise or incorrectly tracked puncta. For example, when a punctum from another filopodium briefly crosses paths, or a punctum that is detected by TrackMate much later than its initial appearance.*

4. Use “Fig4_VelocityAnalysis.Rmd” to process the TrackMate csv and obtain velocities per trajectory. Set paths to the correct TrackMate csv files.

5. Filter out incorrectly segmented puncta, e.g., background noise.

*Note: Keep in mind that if TrackMate analysis included merging and splitting events, sometimes two spots with the same cell identifier and track ID can exist within the same frame. Therefore, the time difference between subsequent frames would equal 0, resulting in division over zero for the speed calculation. To simplify velocity analysis, the code excludes any trajectories containing Myo10 puncta merging or splitting events.*

## Validation of protocol

This protocol has been used and validated in the following research article:

• Shangguan and Rock [14] Hundreds of myosin 10 s are pushed to the tips of filopodia and could cause traffic jams on actin. eLife (Figures 1–4, Figure 1 supplement 1, Figure 2 supplements 1–2, Figure 3 supplements 1–3, Figure 4 supplement 1).

In this work, 150 cells from three bioreplicates were analyzed for the fixed cell experiments, and 30 cells from 3 bioreplicates were analyzed for the live cell experiments.

## General notes and troubleshooting

**General notes**

1. If one is imaging thick cells, confocal microscopy and a full z-stack collection may be needed. Using an epifluorescence microscope works best for imaging flat structures. Flat cells like U2OS are almost entirely within the depth-of-focus, so the entire fluorescence signal is captured by the objective. Even for areas near the nucleus (the thickest point), the out-of-focus signal is blurred within the cell outline. For thick cells (e.g., nonadherent cells with an approximately spherical shape), out-of-focus light can appear beyond the cell boundary and would not be integrated in image processing. These cells require confocal imaging with a z-stack that covers the entire volume of the cell.

2. To count endogenous protein levels, one can conduct CRISPR editing of the native locus of the protein of interest and assess via western blotting.

3. The calmodulin plasmid was originally used in early protocols to ensure that sufficient calmodulin is available in the cells to bind to the Myo10 IQ domains. We have not noticed any significant phenotypic difference when omitting calmodulin in U2OS cells.

4. This protocol quantifies Myo10 by comparing fluorescence intensities to a HaloTag standard protein. However, molecule estimates could be affected by factors such as variations in cellular environment or photobleaching during microscopy, so the user should be aware of these limitations.

5. The image analysis pipeline in this manuscript serves as a template for user customization and is not minimized for computational cost. Furthermore, image segmentation quality could vary between cells and could be improved with pre-processing techniques such as denoising, which is not optimized in these scripts.

## Supplementary information

The following supporting information can be downloaded here:

1. File S1. Nucleotide sequence of HaloTag-Myo10-Flag plasmid.
